# Timing and frequency of antenatal care visits in relation to maternal respiratory syncytial virus vaccine opportunities in four Latin American countries

**DOI:** 10.1016/j.vaccine.2026.128854

**Published:** 2026-08-13

**Authors:** Jessica A. Fleming, Valentina Colistro, Sophia Knudson, Mercedes Colomar

**Affiliations:** a Center for Vaccine Innovation and Access, PATH, 437 N 34th St, Seattle, WA 98103, United States; b Faculty of Medicine, University of the Republic of Uruguay, Avenida General Flores 2125, 11800 Montevideo, Uruguay; c Women's, Maternal, Neonatal and Reproductive Health, Health Systems and Services, Panamerican Health Organization, 525 23^rd^ St NW, Washington, DC 20037, United States

**Keywords:** Maternal immunization, Antenatal care, Respiratory syncytial virus, Generic study protocol, Maternal vaccine, Vaccine implementation, Low- and middle-income countries, Antenatal care timing

## Abstract

Introduction: The Latin American and Caribbean region (LAC) has historically been an early adopter of new vaccines, including the recently introduced maternal vaccine to protect infants against respiratory syncytial virus (RSV). Since maternal RSV vaccination requires gestational age (GA)-specific administration, antenatal care (ANC)-based delivery feasibility depends on ANC visit timing and frequency. We assessed ANC attendance relative to recommended vaccination windows in four LAC countries to inform planning for maternal RSV vaccine implementation and future maternal immunization programs.

Methods: We conducted a retrospective, cross-sectional analysis from Perinatal Information System data across eight tertiary facilities in Bolivia, Ecuador, Honduras, and Uruguay. Women 15–45 years old with ≥1 ANC visit in 2023 and a live, singleton birth at a study facility were included. GA per visit was extracted or derived from last menstrual period or birth data. We defined RSV vaccination eligibility according to Pan American Health Organization (PAHO) (32–36 weeks) and World Health Organization (WHO) (≥ 28 weeks) recommendations. Descriptive analyses summarized ANC initiation, visit timing, and the proportion of women attending visits within eligibility windows.

Results: We included 37,604 women. The median GA at ANC initiation was 12 weeks, ranging from 10 weeks (Uruguay) to 15 weeks (Ecuador). Overall, 63.5% of women attended at least one ANC visit during the PAHO window, with country-specific proportions from 53.6% (Honduras) to 82.3% (Uruguay). Attendance within the WHO window was higher (91.4%). Median ANC attendance was five visits, ranging from five (Honduras) to eight (Uruguay). Earlier ANC initiation was associated with higher visit counts.

Discussion: There is substantial potential for achieving high maternal RSV vaccine coverage in LAC, particularly per broader WHO vaccine recommendations. Where ANC begins later or visits are fewer, narrower vaccine-eligible windows may limit achievable coverage. Strengthening early ANC engagement and immunization counseling may increase vaccination opportunities.

## 1 Introduction

Under Pan American Health Organization (PAHO) guidance, the Latin American and Caribbean region (LAC) has a long-standing history of early adoption of new vaccines, including vaccines recommended during pregnancy [1], [2], [3]. Argentina was the first country worldwide to introduce a new respiratory syncytial virus (RSV) vaccine in pregnant women in 2024 [4], [5]. Subsequently in the region, Brazil, Chile, and Uruguay introduced the maternal RSV vaccine into their national immunization programs [6], [7], [8]. RSV is the leading cause of severe lower respiratory tract infections (including bronchiolitis and pneumonia) and hospitalizations in infants globally [9], [10], [11]. Through antibodies transferred across the placenta, the maternal RSV vaccine protects young infants from RSV disease in the first months after birth, when infants are most at risk.

Maternal vaccines are commonly delivered through antenatal care (ANC) services. While some maternal vaccines, such as tetanus toxoid–containing vaccines, may be administered at any point during pregnancy, others require more precise timing. The maternal RSV vaccine is recommended for use within a specified gestational age (GA) window, which varies by regulatory authority, reflecting differences in risk-benefit assessments for intended populations and implementation considerations. For example, both PAHO and the US Centers for Disease Control and Prevention recommend vaccination between 32- and 36-weeks' gestation, while the World Health Organization's (WHO's) Strategic Advisory Group of Experts on Immunization recommends administration during the third trimester, as locally defined [12], [13], [14]. Several maternal vaccines currently in development, including candidates targeting Group B *Streptococcus*, are also expected to require similarly defined gestational windows for administration. For ANC services to serve as an effective vaccine delivery platform with such timing restrictions, attendance of ANC visits must align with recommended immunization windows.

Countries considering the introduction of new vaccines often draw on experiences from LAC to guide policy development. In this study, we evaluate the number and timing of women's attendance at ANC visits relative to maternal RSV vaccine eligibility windows using data from a retrospective, cross-sectional analysis conducted in eight tertiary-level health facilities in Bolivia, Ecuador, Honduras, and Uruguay. By characterizing ANC attendance patterns during PAHO- and WHO-recommended immunization windows, our findings can inform decision-making and operational planning for maternal RSV vaccine introduction within LAC more broadly and offer insights relevant to maternal immunization programs globally.

## 2 Material and methods

PAHO and PATH conducted this retrospective, cross-sectional study in 2024 and 2025 using a generic study protocol, data collection tool, and data analysis plan developed by PATH in collaboration with WHO (see *Supplementary Material 1*). Tools were adapted for electronic data extraction. The study was part of a broader multi-country initiative supported by WHO, which also included studies in Mozambique and Việt Nam; results will be reported separately.

The study facilities constitute a network of sentinel health centers in LAC coordinated by the Women's, Maternal, Neonatal and Reproductive Health Unit (WH) within PAHO's Department of Health Systems and Services (HSS). Sentinel facilities in Bolivia, Ecuador, Honduras, and Uruguay were included; network sites in Argentina and Honduras were excluded due to incomplete data availability.

Anonymized data were obtained from the Perinatal Information System (SIP), a digital clinical records platform coordinated by WH/HSS that captures standardized demographic, diagnostic, obstetric, and neonatal outcomes across pregnancy [15]. The SIP aims to aggregate information on ANC visits conducted at all health facilities within the respective country, allowing for comprehensive documentation of maternal health care utilization throughout pregnancy.

We included records of pregnancies from women 15 to 45 years of age with at least one recorded ANC visit in 2023 and a live, singleton birth at one of eight tertiary-level sentinel facilities. Extracted variables included maternal age, date of ANC visit, and GA (in weeks) at each ANC visit. Additional variables were recorded and used to calculate GA for visits with missing GA values, including date of last menstrual period (LMP), infant date of birth, and GA at birth as assessed by an obstetrician or neonatologist. All ANC visits for each participant were included. In Bolivia, data were available for a maximum of seven visits per pregnancy. We excluded women with missing age, any ANC visit recorded at >44 weeks' GA, and women with any ANC visit lacking sufficient data to derive GA. We rounded GA values down to the nearest whole week for analysis.

We determined maternal RSV vaccine eligibility at each ANC visit according to whether the GA at the visit aligned with vaccine administration recommendations from PAHO (32 to 36 weeks' gestation) and/or WHO (any time during the third trimester). Trimesters were defined using Global Alignment of Immunization Safety Assessment (GAIA) criteria: first trimester, 1 to 13 weeks; second trimester, 14 to 27 weeks; and third trimester, ≥ 28 weeks [16].

We conducted all analyses using R Statistical Software (version 4.3; R Foundation for Statistical Computing, Vienna, Austria). Ethical exemption for this study was granted by WCG IRB and the PAHO Ethical Review Committee.

## 3 Results

### 3.1 Population characteristics and timing of ANC initiation

Analyses included 184,611 ANC records from 37,604 women. Honduras contributed the highest proportion of the study population (62.9%) (Table 1). The mean age of women initiating ANC was 25.4 years. Overall, the median GA at the first ANC visit was 12 weeks, ranging from 10 weeks in Uruguay to 15 weeks in Ecuador.

**Table 1:** Characteristics of pregnant women attending antenatal care by country.

	Country									
	Bolivia		Ecuador		Honduras		Uruguay		All	
Characteristics	N	%	N	%	N	%	N	%	N	**%**
Facilities	2	25.0	1	12.5	3	37.5	2	25.0	8	100.0
Pregnant women	4364	11.6	4730	12.6	23,641	62.9	4869	12.9	37,604	100.0
ANC records	23,399	12.7	27,917	15.1	96,167	52.1	37,128	20.1	184,611	100.0
Mean age, years	26.2		26.3		25.1		25.7		25.4	

Gestational age in weeks at first ANC visit										
Mean (SD)	15.6 (8.2)		16.9 (8.5)		15.3 (9.2)		11.6 (6.5)		15.0 (8.8)	
Median	14		15		12		10		12	
Min–Max	1–40		2–40		1–44		2–39		1–44	

ANC = antenatal care; Max = maximum; Min = minimum; N = number; SD = standard deviation

### 3.2 ANC attendance by maternal RSV vaccination recommendations

**Fig. 1:**
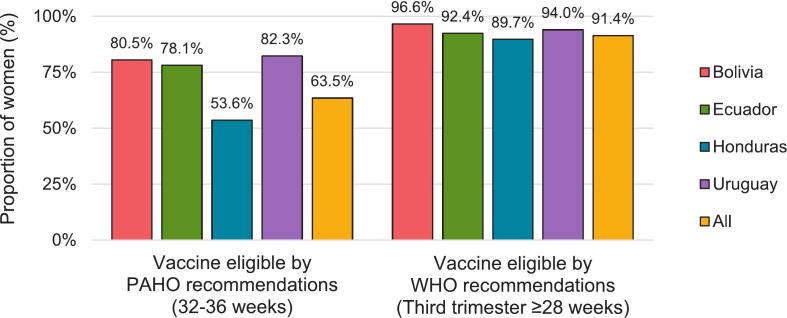
Women attending antenatal care at least once within RSV vaccine eligibility window, by country (%).

Across all countries, 63.5% of women attended at least one ANC visit during the PAHO-recommended maternal RSV vaccination eligibility window (32 to 36 weeks GA); attendance within this window was highest in Uruguay (82.3%) and lowest in Honduras (53.6%) (Fig. 1). Using the broader WHO maternal RSV vaccination recommendation period of third trimester defined as ≥28 weeks gestation, overall attendance was 91.4%, ranging from 96.6% in Bolivia to 89.7% in Honduras. Across all countries, most women attended at least one ANC visit before becoming eligible for RSV vaccination (92.9% and 88.0%, according to PAHO and WHO recommendations, respectively). (For ANC attendance by additional windows, see Table 1, *Supplementary Material 2*).

### 3.3 Timing and frequency of ANC visits across pregnancy

**Fig. 2:**
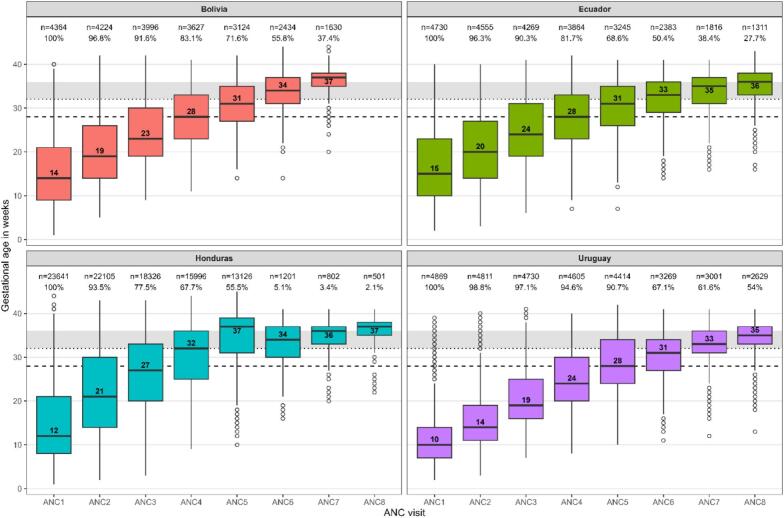
Median and interquartile range of gestational age in weeks at antenatal care visits by visit order through eight visits^1^, by country. ^1^Bolivia records included up to seven visits. ANC1 = first ANC visit; ANC2 = second ANC visit, etc.; ANC = antenatal care. Box plot describing the gestational age in weeks by ANC visit number (first through eighth [seventh in Bolivia]). The solid line within each box represents the median gestational age (in weeks) of the visit; also indicated by the number at the top of the box. The proportion of women attending each visit is also shown. The dotted line represents the beginning of the PAHO maternal RSV vaccine recommendation of 32 weeks gestation; the grey band indicates the PAHO eligibility window of 32 to 36 weeks gestation; the dashed line represents the beginning of the WHO-recommended eligibility window of third trimester, using GAIA's definition of 28 weeks gestation.

Over half of our study population (55.7%) made at least one ANC visit in the first trimester of pregnancy, 79.9% in the second trimester, and 91.4% in the third trimester (Table 2, *Supplementary Material 2*). Uruguay had the highest coverage of women attending four or more ANC visits per pregnancy (94.6%), followed by Bolivia (83.1%), Ecuador (81.7%), and Honduras (67.7%) (Fig. 2). The highest coverage of eight or more visits per pregnancy was in Uruguay (54.0%). Ecuador was 27.7% and Honduras was 2.1%. Coverage of women attending at least seven visits per pregnancy in Bolivia (the maximum recorded in the SIP database for Bolivia) was 37.4%.

**Table 2:** Antenatal care visits per woman by gestational age window.

	ANC visits per woman		
Gestational age window	Mean (SD)	Median	Min-Max
Anytime (1–44 weeks) [all countries]	4.9 (2.3)	5	1–22
Bolivia^1^	5.4 (1.7)	6	1–7
Ecuador	5.9 (2.7)	6	1–22
Honduras	4.1 (1.6)	5	1–15
Uruguay	7.6 (2.8)	8	1–15
Trimester 1 (1–13 weeks) [all countries]	0.8 (0.9)	1	0–8
Trimester 2 (14–27 weeks) [all countries]	1.8 (1.3)	2	0–11
Trimester 3 (≥ 28 weeks) (WHO RSV vaccine recommendation) [all countries]	2.3 (1.5)	2	0–14
32–36 weeks (PAHO RSV vaccine recommendation) [all countries]	0.9 (0.9)	1	0–6

1 Bolivia records included up to seven visits. ANC = antenatal care; Min = minimum; Max = maximum PAHO = Pan American Health Organization; RSV = respiratory syncytial virus; SD = standard deviation.

In Honduras, a median of 32 weeks of gestation (the beginning of maternal RSV vaccine eligibility in PAHO) was reached at the fourth ANC visit, while this was reached in Bolivia and Ecuador at the sixth visit and at the seventh visit in Uruguay (Fig. 2, dotted line). A median GA of 28 weeks of gestation (the beginning of vaccine eligibility according to WHO guidelines based on GAIA trimester definitions) was met by the fourth visit in Bolivia, Ecuador, and Honduras and by the fifth visit in Uruguay (Fig. 2, dashed line).

Across all countries, women attended a median of five ANC visits per pregnancy, ranging from five visits in Honduras to eight visits in Uruguay (Table 2). Before vaccine eligibility, women made a median of one visit during the first trimester and two visits during the second trimester. Women attended a median of one visit within the PAHO-recommended maternal RSV vaccination window and two visits within the WHO-recommended window.

**Fig. 3:**
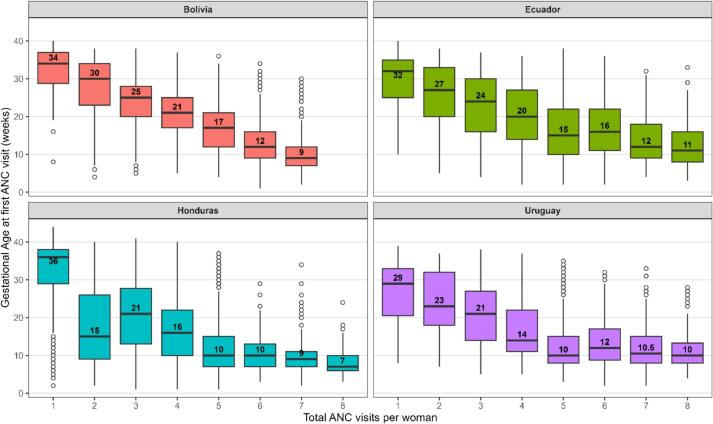
Gestational age at first antenatal care visit by total number of visits^1^, by country. ^1^Bolivia records included up to seven visits. 1 = total of one visit during pregnancy; 2 = total of two visits during pregnancy, etc.; ANC = antenatal care. Box plot describing the gestational age in weeks at first ANC visit by total number of ANC visits. The solid line within each box and the number above the line represent the median gestational age (in weeks) of women attending the respective number of ANC visits in pregnancy.

Overall, women attending a greater number of ANC visits began care earlier in pregnancy (Fig. 3). Women with one visit had a median GA at that visit of 35 weeks; whereas, women with four visits initiated ANC at a median GA of 17 weeks (composite results not shown). Women with eight recorded visits initiated care earlier, with a median GA of 10 weeks. Similar patterns of earlier ANC initiation with increasing visit frequency up to eight visits in Ecuador, Honduras, and Uruguay and up to seven visits in Bolivia were observed across all countries (Fig. 3).

## 4 Discussion

Argentina and Uruguay were among the first countries to introduce the maternal RSV vaccine into their public health programs and other countries in the region may soon consider maternal immunization for the prevention of RSV in young infants. This study aimed to evaluate the timing and frequency of ANC visits and assess their alignment with PAHO and WHO maternal RSV vaccination recommendations to estimate the potential vaccine coverage achievable through ANC-based delivery. Although several studies [17], [18], [19] have characterized ANC visit timing in relation to maternal vaccine administration, to our knowledge, this is the first to report such findings from LAC—a region with a longstanding history of early vaccine adoption and significant influence on immunization practices in other regions. As part of a larger multi-site effort, this analysis is among the first to evaluate ANC visit timing relative to the gestational age window recommended by WHO for RSV vaccination during pregnancy.

We found that 64% of women across our study countries attended at least one ANC visit during the maternal RSV vaccination window recommended by PAHO (32 to 36 weeks' gestation) and, therefore, would have been eligible to receive the vaccine during this period, providing an estimate of potential vaccination coverage. This overall proportion is comparable to the 62.5% coverage estimate used in a maternal vaccine effectiveness study conducted in Argentina in 2024 [20]. We observed, however, substantial variation in potential vaccination coverage across countries, ranging from 54% in Honduras to 78% in Ecuador, 81% in Bolivia, and 82% in Uruguay. Our finding is higher than the 47% estimated in a multi-country analysis from Bangladesh, The Gambia, Nepal, Nigeria, Tanzania, and Uganda [17]—derived from interpolation of the timing of first and last ANC visits—but it nonetheless indicates a gap in potential vaccine coverage. Applying the broader WHO recommendation of administering the vaccine at any time during the third trimester [21] has the potential to substantially increase coverage given that 91% of women in our study attended at least one ANC visit during this expanded eligibility window. Important to note, however, is that this finding may overestimate potential population-level coverage, as it is derived solely from women that accessed ANC services. It therefore excludes women that did not engage with the health system, including maternal immunization programs, and does not account for vaccine acceptance.

Although WHO recommends third-trimester vaccination to maximize antibody transfer and neonatal protection, PAHO adopted a more conservative 32 to 36-week window [22]. This decision was informed by study findings showing that subgroup analyses of non–high-income countries suggested a possible increase in preterm birth risk when vaccination occurred earlier [23], [24]. While this evidence remains inconclusive, it is relevant for the LAC region, which includes several low- and middle-income countries (LMICs).

In 2016, WHO published updated guidelines on care in pregnancy, which recommends eight ANC visits per pregnancy [25]. Although 75% of women in Ecuador, Honduras, and Uruguay attended at least four ANC visits per pregnancy, only 12% completed the full eight-visit schedule recommended by WHO. The median number of ANC visits per pregnancy in our study population was five, slightly higher than the median of four visits per pregnancy recently reported by Ford et al. from Bangladesh, The Gambia, Nepal, Nigeria, Tanzania, and Uganda [17]. Uruguay was the lone country in our study classified by the World Bank as high-income [26] and for which the median number of visits per pregnancy met the WHO recommendation of eight visits, indicating a persistent gap in service provision in the other countries. In Bolivia, the data system records a maximum of seven visits, so adherence to the full schedule could not be assessed.

While only 56% of our study population met WHO's recommendation for initiating prenatal care during the first trimester of pregnancy [25], 88% attended at least one ANC visit during the first or second trimester, before eligibility for maternal RSV vaccination by either PAHO or WHO recommendations. Besides providing a host of other critical ANC services, these early visits are important opportunities for health workers to educate and sensitize women about maternal immunization, which may influence vaccine uptake later in pregnancy. We report an overall median GA at the first ANC visit of 12 weeks, within the first trimester, but found differences across countries, with median first visits as early as 10 weeks in Uruguay to as late as 15 weeks in Ecuador [27]. These are considerably earlier than Nyiro et al. reported in a study of women attending ANC screening in Kenya, where the median GA at ANC initiation was 26 weeks [28], [29]. Pay-for-performance programs in LAC (e.g., such as Uruguay's *Metas Asistenciales* [Healthcare Goals] that provides economic incentives to health care institutions for complying with certain maternal and newborn health care goals) have been associated with both an increase in women attending the recommended number of ANC visits and earlier ANC initiation in pregnancy [27]. In LMICs, the sustainability of such programs may present challenges.

Similar to Ford et al., we found that women initiating care earlier made more visits overall. These findings underscore the need for enhanced health worker education and community engagement efforts to promote earlier and more frequent ANC attendance and sustained participation throughout pregnancy.

We found that most women initiated ANC during the second trimester and attended only one visit within the PAHO-recommended eligibility window for maternal RSV vaccination, resulting in a single opportunity for vaccine administration. When applying the broader WHO-recommended vaccination window, the number of potential vaccination opportunities doubled, with women attending a median of two ANC visits during this period. These findings underscore the importance of further research to better characterize potential benefits and risks of broadening the administration recommendation, particularly considering that more opportunities for vaccination would be available if the WHO-recommended window were adopted.

Our study has multiple strengths. The use of a shared clinical record system from institutions in multiple countries via the SIP database enabled inclusion of all women that sought care during the study period, without having to randomize inclusion and minimized selection bias. The database is comprehensive, capturing multiple variables to determine the GA at each visit. It also records the complete visit histories of women, including those attending other health facilities in the respective study countries, minimizing the risk of missing data.

Our study also has several limitations. First, the SIP database in Bolivia included data from a maximum of seven ANC visits. This restricted the scope of analyses for Bolivia and underscored persistent challenges in achieving compliance with the WHO recommendation of eight ANC visits per pregnancy. Second, when GA records were unavailable, we estimated GA using an algorithm based on LMP or infant date of birth rather than ultrasound records. While ultrasound is a more precise method when conducted early in pregnancy, the ultrasound data in SIP did not include the GA at which it was performed. Finally, we lacked information on women that did not access the health system—a population whose infants may be at increased risk for RSV infection and related pregnancy complications.

This study provides evidence on the timing and frequency of ANC visits in relation to maternal RSV vaccine eligibility in four LAC countries, offering practical insights for planning ANC-based vaccine delivery. Our findings demonstrate substantial potential for achieving high coverage within the broader WHO-recommended gestational window, while also highlighting gaps in ANC utilization. Overall, this work strengthens the evidence base needed to inform efficient maternal immunization strategies and supports countries as they consider integrating RSV and future maternal vaccines into routine ANC services.

## CRediT authorship contribution statement

**Jessica A. Fleming:** Conceptualization, Formal analysis, Funding acquisition, Investigation, Methodology, Project administration, Supervision, Validation, Writing – original draft, Writing – review & editing. **Valentina Colistro:** Data curation, Formal analysis, Writing – review & editing. **Sophia Knudson:** Formal analysis, Methodology, Project administration, Visualization, Writing – review & editing. **Mercedes Colomar:** Investigation, Methodology, Project administration, Writing – review & editing.

## Disclaimer

Authors who are staff members of the PAHO are solely responsible for the opinions expressed in their texts, which may not necessarily reflect the opinion or policy of the PAHO.

## Funding

This work was supported by the 10.13039/100004423WHO.

## Declaration of competing interest

The authors declare the following financial interests/personal relationships which may be considered as potential competing interests: Jessica A. Fleming reports financial support was provided by World Health Organization. Valentina Colistro reports financial support was provided by PATH. Sophia Knudson reports financial support was provided by World Health Organization. Mercedes Colomar reports financial support was provided by PATH. If there are other authors, they declare that they have no known competing financial interests or personal relationships that could have appeared to influence the work reported in this paper.

## Data Availability

The authors do not have permission to share data.

## Appendix A Supplementary data

Supplementary Material 1: Generic study protocol and data analysis plan.
