# Supplementary material for: Timing and frequency of antenatal care visits in relation to maternal respiratory syncytial virus vaccine opportunities in four Latin American countries
Source: Vaccine. 2026 Aug 13;88:None. doi: 10.1016/j.vaccine.2026.128854 (PMC13481105; doi:10.1016/j.vaccine.2026.128854)
Supplement: Supplementary file 1 [file mmc1.docx]

**Supplementary Material 1**

Supplementary Material 1a. Study protocol

**Characterizing the timing and attendance of antenatal care visits to inform the delivery of new maternal vaccines in low- and middle-income countries**

Generic ANC Timing Study Protocol

PATH

17 November 2025

Table of Contents

[List of Abbreviations	4](#_Toc213246849)

[Protocol Summary	4](#_Toc213246850)

[1.0	Introduction	5](#_Toc213246851)

[2.0	Background	5](#_Toc213246852)

[3.0	Study objective and endpoint	6](#_Toc213246853)

[4.0	Methods	6](#_Toc213246854)

[4.1	Study design and data sources	6](#_Toc213246855)

[4.2	Data collection tools and software	6](#_Toc213246856)

[4.3	Health facility selection	7](#_Toc213246857)

[4.3.1 Sampling strategy	7](#_Toc213246858)

[4.3.2 Inclusion criteria for health facilities	8](#_Toc213246859)

[4.3.3 Exclusion criteria for health facilities	8](#_Toc213246860)

[4.4	Study period selection	9](#_Toc213246861)

[4.5	Participant selection	9](#_Toc213246862)

[4.5.1	Study population	9](#_Toc213246863)

[4.5.2	Inclusion criteria	9](#_Toc213246864)

[4.5.3	Exclusion criteria	9](#_Toc213246865)

[4.5.4	Sampling strategy and randomization approach	9](#_Toc213246866)

[4.5.5	Documenting ineligible individuals	9](#_Toc213246867)

[4.6	Study variables	10](#_Toc213246868)

[4.6.1	Facility-level data	10](#_Toc213246869)

[4.6.2	Individual-level data	10](#_Toc213246870)

[5.0	Ethical considerations	11](#_Toc213246871)

[6.0	Data collection quality control	11](#_Toc213246872)

[7.0	Data management	12](#_Toc213246873)

[7.1	Confidentiality	12](#_Toc213246874)

[7.2	Data storage and access	12](#_Toc213246875)

[7.3	Quality control measures	12](#_Toc213246876)

[7.4	Handling missing data	13](#_Toc213246877)

[7.5	Handling data inconsistencies	13](#_Toc213246878)

[8.0	Data analysis	13](#_Toc213246879)

[8.1	Statistical software	14](#_Toc213246880)

[8.2	Descriptive analysis	14](#_Toc213246881)

[9.0	Acknowledgements	14](#_Toc213246882)

List of Abbreviations

| ANC | Antenatal care |
| --- | --- |
| ANC1 | First antenatal care visit |
| ANC4 | Fourth antenatal care visit |
| EDD | Estimated date of delivery |
| FH | Fundal height |
| GA | Gestational age |
| GBS | Group B *Streptococcus* |
| IRB | Institutional Review Board |
| LMICs | Low- and middle-income countries |
| LMP | Last menstrual period |
| RSV | Respiratory syncytial virus |
| US | Ultrasound |
| WHO | World Health Organization |

Protocol Summary

| **Title** | Characterizing timing and attendance of antenatal care visits to inform the delivery of new maternal vaccines in low- and middle-income countries |
| --- | --- |
| **Short title** | ANC Timing |
| **Protocol number** | Version 2.0 |
| **Study objective** | Characterize the distribution of antenatal care (ANC) visits by gestational age (GA) |
| **Study endpoint** | Proportion of individuals attending ANC within specified GA windows corresponding to vaccine eligibility |
| **Study design** | Retrospective cross-sectional |
| **Data sources** | Secondary, quantitative data from paper-based ANC registers organized by individual from health facilities across levels of care |

1.0 Introduction

This generic protocol provides a high-level description of study procedures to characterize the timing and attendance of antenatal care (ANC) visits to inform the delivery of new maternal vaccines in low- and middle-income countries (LMICs) that have specific vaccination timing recommendations. These tools are publicly available and may be freely used or adapted for other studies. Designed to be applicable to any maternal vaccine and suitable for implementation across diverse settings, investigators can modify the materials to align with specific maternal vaccine administration schedules and local health system contexts. The study uses a retrospective design, which requires fewer resources and less time to implement than a prospective approach while still providing valuable insights into ANC attendance patterns relevant to maternal vaccine delivery.

This protocol is part of a set of study materials that includes a pre-programmed XLS Form to upload to the open-source software **Kobo Toolbox** for the **ANC Timing Data Collection Tool** and an **ANC Timing Data Analysis Plan**.

The objective of this study is to characterize the distribution of ANC visits by gestational age. To achieve this objective, the study will record the dates of ANC visits that individuals made within the study period and document the respective gestational ages (GA) of each visit. The key analysis is to identify the visits that were made at GAs in which individuals were eligible to receive a given maternal vaccine, as per a defined GA range. A primary definition of vaccine eligibility will be defined *a priori*, however various windows can be explored.

2.0 Background

New vaccines have been licensed, and others are in development, specifically for use in pregnancy to enhance immunity for mothers and protect their babies against infectious diseases in early life. Among these maternal vaccines now licensed and recommended by the World Health Organization (WHO) is a vaccine against respiratory syncytial virus (RSV)—an important cause of respiratory infection in young children. Other vaccine candidates for Group B *Streptococcus* (GBS) (the leading cause of early life invasive bacterial disease) and other pathogens, are not far behind. Introduction readiness and success for these new vaccines in LMICs will depend, in part, on immunization and ANC program coordination to ensure that vaccines reach pregnant individuals at optimal gestation periods to maximize immunity and protection.

Vaccines against tetanus have safely and effectively been given during pregnancy in LMICs for decades; however, introducing new vaccines developed and indicated specifically for use in pregnancy in these contexts presents opportunities and challenges beyond those associated with traditional maternal tetanus immunization strategies. While tetanus vaccine can be given to individuals during any trimester of pregnancy, recommendations for new maternal vaccines, such as RSV vaccines, indicate specific windows of administration defined by GA and may differ by location. For example, the United States and WHO’s Pan American Health Organization recommend maternal RSV vaccination between 32- and 36-weeks gestation. Europe and the United Kingdom respectively recommend use between 24 and 36 weeks and from 28 weeks to birth. In September 2024, WHO’s Strategic Advisory Group of Experts on Immunization recommended maternal RSV vaccine administration in the third trimester of pregnancy, as defined locally.^[1]^

Effective planning for maternal vaccine introduction requires a clear understanding of ANC attendance patterns, including the timing and frequency of visits and the extent to which these align with recommended vaccination windows. This study will provide information about when and how often individuals access ANC services. This information can inform maternal health services and vaccine delivery efforts, including optimizing coverage and improving assessment of risks and benefits of newly introduced maternal vaccines.

3.0 Study objective and endpoint

This study will count the number of ANC visits that individuals made within the study period and document the respective GAs of each visit. Specific vaccine eligibility gestational age ranges, or windows, will then be applied to identify the visits in which individuals were eligible to receive a specific maternal vaccine.

**Primary objective**	Characterize the distribution of ANC visits by GA.

**Primary endpoint**	Proportion of individuals attending ANC within specified GA windows corresponding to vaccine eligibility.

4.0 Methods
    4.1 Study design and data sources

This is a **retrospective study**. It uses a **cross-sectional methodology using secondary data**. Basic **facility-level data** are collected from all study facilities and **individual-level data** are collected from a random sample of individuals. Variables include the date of each of the individual’s ANC visits and the corresponding GAs at each visit. These data can be collected from paper-based ANC registers organized by individual maintained by study facilities for a retrospective period of 12 consecutive months.

4.2 Data collection tools and software

Data will be collected using a pre-programmed digital **ANC Timing Data Collection Tool** using the open-source software **Kobo Toolbox**. A standardized data collection tool in the format of an XLS form is included in the **ANC Timing** **Data Collection Tool** supplementary material that can be uploaded to Kobo Toolbox.

4.3 Health facility selection

4.3.1 Sampling strategy

A **purposive sampling** approach will be used to select health facilities for inclusion in the study. The aim is to include a diverse and informative sample of facilities that represent the range of health system contexts in which maternal immunization is/may be administered within the country.

When determining the number and geographic distribution of facilities, considerations should include the following:

- **Diversity in health facility types**, such as
  - Levels of care (e.g., **primary**, **secondary**, **tertiary**);
  - Type of facility (e.g., **public**, **private**); and
  - ANC clinic volume (e.g., **number of ANC visits/month, catchment area, population size)**
- **Diversity in public health performance metrics**, such as
  - Average timing of first ANC visit;
  - Coverage of four or more ANC visits;
  - Proportion of facility-based deliveries; and
  - Coverage of maternal tetanus toxoid-containing vaccines.
- **Diveristy in sociodemographic characteristics** of the catchment population, such as
  - **Urban/rural** classification; and
  - **Economic status** of the area (e.g. average household income, literacy).

This sampling approach is intended to maximize the generalizability of study findings to the national context, while also maintaining the logistical feasibility of data collection.

Site selection example:

1. Select two provinces
   - Maximize diversity with respect to available sociodemographic indicators (e.g., urban/rural, average household income, literacy).
2. Select two districts in each province
   - Include one high-performing and one low-performing district in each province based on health system performance using available information (e.g., average timing of first ANC visit, percent coverage of four or more ANC visits, percent of facility-based deliveries, percent coverage of maternal tetanus toxoid-containing vaccine).
3. Select two primary, one secondary, and one tertiary health facility in each district.
4. If the private sector is an important source of ANC service provision at a given level of care in a selected district, select at least one private health facility at that respective level of care.

4.3.2 Inclusion criteria for health facilities

**This protocol assumes that health facilities maintain paper-based ANC records**. A health facility with paper-based records will be eligible for inclusion in the study if it meets the following criteria:

- Facility provides **routine ANC** to pregnant individuals.
- Facility provides **routine** **immunizations** to pregnant individuals.
- Facility maintains **ANC records organized by individual** (i.e., each individual's visits across an index pregnancy are grouped under a single entry) rather than by visit date (i.e., all individuals making a visit on a given day are grouped under a single date).
- **Facility ANC registers must document the following**:
  - **Date of first ANC visit** and all **subsequent visits**.
  - **GA** or at least one proxy variable that allows for GA estimation, such:
    - Date of **last menstrual period (LMP)**
    - **Estimated delivery date (EDD)**
    - Date of **ultrasound (US)** and corresponding GA
    - **Fundal height (FH)**
- Facility ANC records must be **available for a consecutive 12-month period** during the study timeframe.
- Facility ANC registers must be accessible and facility staff available to respond to questions regarding data completeness or structure.

4.3.3 Exclusion criteria for health facilities

Health facilities will be **excluded** from the study if any of the following conditions apply:

- Facility does **not provide routine ANC** to pregnant individuals (e.g., specialized clinics, referral-only facilities).
- Facility does **not provide routine immunizations** to pregnant individuals.
- Facility does **not maintain individual-level ANC records** or records are **incomplete or unavailable** for the 12-month study period.
- Facility with an ANC register that does not collect the **date of the first ANC visit** and **subsequent visits** and does not collect **at least one variable that can be used to** **estimate** **GA**.
- Facility lacks available **key staff** that can provide access to ANC registers and respond to questions regarding data completeness or structure.
  4.4 Study period selection

Select a **retrospective and consecutive 12-month study period** in which all study facilities have ANC registers available for data extraction. Avoid periods with widespread and/or acute disruptions (e.g., a pandemic, prolonged health personnel strikes, or facility construction) that may have affected ANC services or the likelihood of individuals attending health services.Top of Form

4.5 Participant selection
    4.5.1 Study population
- The study population will consist of pregnant individuals with records of attending ANC visits at selected study health facilities during a defined retrospective 12-month study period.
  4.5.2 Inclusion criteria
- Individuals with a record of attending ANC at one of the selected study health facilities.
- Individuals with a singleton pregnancy.
  4.5.3 Exclusion criteria
- Individuals with index pregnancy ending in miscarriage, abortion, or stillbirth.
- Individuals with index pregnancy that is a multiple pregnancy (e.g., twins, triplets).
  4.5.4 Sampling strategy and randomization approach

Select a **random sample of eligible individuals** at each health facility **over the retrospective 12-month study period** to ensure even temporal distribution and to minimize seasonal or monthly bias in ANC attendance patterns. The sampling fraction should consider the **size of the facility** and the **feasibility** and **affordability** of data collection. Example sampling fractions include 1 out of every 2 individuals, 1 out of every 5 individuals, or 1 out of every 25 individuals. Either individual or block randomization can be used.

4.5.5 Documenting ineligible individuals

Individuals randomly selected for inclusion in the study, but not meeting the eligibility criteria, should be recorded on an ineligibility table such as the one shown in Table 1. Investigators may revise this table to address any additional eligibility requirements relevant to the local context.

Table 1. Ineligibility table

| Ineligible individual number | Health facility name | Date of record review | Ineligibility criteria (mark all that apply) | | | Comments, if any |
| --- | --- | --- | --- | --- | --- | --- |
|  |  |  | Missing date of initial visit | Missing gestational age | Multiple pregnancy, miscarriage, abortion, stillbirth |  |
| 1 |  |  |  |  |  |  |
| 2 |  |  |  |  |  |  |
| 3 |  |  |  |  |  |  |

4.6 Study variables

Data will be collected at both the **facility level** and the **individual level** using a standardized data collection tool. Every effort should be made to collect as many of the listed variables as are available and relevant within the local context, noting that the name of each indicator may differ based on local context.

4.6.1 Facility-level data

Prior to initiating individual-level data collection, **facility-level data,** including facility name, location, and level of care, will be obtained through a combination of document review and consultation with facility staff (Table 2). This information will be used to automate the **ANC Timing Data Collection Tool** with facility-level data for each site when a health facility name is selected in Kobo Toolbox. The **ANC Timing** **Data Collection Tool** includes example calculations that are highlighted in the XLS Form. The investigator will need to edit these calculations using the facility-level data below and update the red text with the respective health facility and location names.

Table 2. Health facility variables

| **Variable** | **Description** |
| --- | --- |
| Health facility name | Official name of the health facility. |
| Type of facility | Classification of the health facility (e.g., public, private). |
| Level of care | Level of service provision (e.g., primary, secondary, tertiary). |
| Province | Geographic area in which the facility is located. |
| District | Smaller administrative unit. |
| Town | Location of the health facility. |
| Urbanicity | Categorical measure of the catchment area’s urban or rural status. |
| ANC clinic volume | Average number of ANC visits to the facility per year, month, or day (as available from administrative data), then categorized into small (1–100 visits/month), medium (101–200 visits/month), or large (>200 visits/month). |

4.6.2 Individual-level data

Individual-level data will be extracted from **routine ANC registers** maintained at each selected health facility (Table 3). These data (such as age, date of visit, date of last menstrual period, expected date of delivery, and fundal height) represent routinely collected clinical information and will be abstracted retrospectively using standardized data collection tools adapted to the local context.

Table 3. Individual variables

| **Variable** | **Description** |
| --- | --- |
| Age of pregnant individual (years) | Age of the individual at the time of the first recorded ANC visit. |
| Date of last menstrual period | As recorded in the ANC register; format: YYYY-MM-DD. |
| Expected date of delivery (EDD) | As recorded or estimated by health provider; format: YYYY-MM-DD. |
| **For each ANC visit** | |
| Date of ANC visit | Calendar date of the ANC visit; format: YYYY-MM-DD. |
| Gestational age | GA in weeks at the time of the visit |
| Ultrasound | GA in weeks by ultrasound at the time of the scan |
| Fundal height | Fundal height measured in centimeters (cm), if recorded. |
| Comments | Any relevant notes. |

5.0 Ethical considerations

Obtain ethical approvals from relevant institutional review boards and/or administrative bodies at the country, division, sub-division, and health facility levels, as required, before commencing the study.

6.0 Data collection quality control

Before data collection begins, the randomization and data collection procedures will be pre-tested through a hands-on practice session conducted during training. The objective of this exercise is to ensure that all study personnel understand and can accurately implement the protocol and data recording procedures.

During the initial randomization cycles at each study facility, a supervisor will directly observe and oversee the randomization and data collection processes. Supervisors will verify that procedures are performed in compliance with the protocol and that all entries are complete and consistent with source documentation. Supervisors will provide feedback and corrective guidance to data collectors as necessary to address documentation issues, procedural errors, and protocol deviations.

Throughout the study period, supervisors will conduct end-of-day audits of data to ensure continued compliance and data integrity**.**

7.0 Data management

All data collection, management, quality control, retention, and access procedures will follow applicable institutional, ethical, and funder-specific guidelines and requirements. The study will adhere to best practices in data security, participant confidentiality, and data integrity throughout the data lifecycle.

7.1 Confidentiality

To ensure the confidentiality and integrity of study data,

- Personally identifiable information (e.g., participant names, phone numbers, addresses, and health identification numbers) will **not** be collected.
- Each participant will be assigned a **study-specific unique identifier** to de-identify records.
- Data will only be accessible to **authorized study personnel** and access will be limited to the minimum necessary for each team member’s role.
- Data will be collected, stored, analyzed, and visualized using **password-protected, encrypted devices** and platforms.
- All staff with access to study data must have valid **Human Subjects Research training** certifications on file.
  7.2 Data storage and access

Data will be securely stored using **cloud-based, encrypted platforms** integrated with the **ANC Timing** **Data Collection Tool**.

- **Encrypted storage** will be used to protect data.
- **Access permissions** will be role-based.
  - Data collectors will have access only to enter or review assigned records.
  - Data managers and analysts will have broader access to support data cleaning and analysis.
  7.3 Quality control measures

A series of data quality control procedures will be built into the data collection and management system to ensure **accuracy, reliability, and completeness** per the following:

- **Data entry restrictions**
  - Fields will be configured to accept only valid formats (e.g., YYYY-MM-DD for dates).
  - Mandatory fields will be enforced where critical data are required.
- **Validation criteria**
  - Validation rules may include GA limits, age limits, fundal height ranges, and cross-checks between related fields.
- **Skip logic**
  - Implemented to reduce irrelevant or inconsistent responses (e.g., questions about follow-up visits will only appear if a follow-up visit is recorded).

These system-level checks will minimize errors and reduce the need for extensive post-entry data cleaning.

7.4 Handling missing data

Individuals must have a recorded **date of first ANC visit** and at least one **GA indicator** to be eligible for inclusion in the study.

- If GA is missing for subsequent visits, it will be **estimated** using predefined methods outlined in the **ANC Timing** **Data Analysis Plan** (e.g., based on LMP, EDD, fundal height, or US).
- Missing data for **non-essential variables** will be managed using **standard imputation methods** during analysis.
- Individuals with **missing critical variables** may be excluded from specific analyses, with exclusions clearly documented.
  7.5 Handling data inconsistencies

**During the process of data collection**, the data manager will be responsible for quality. **Quality control must be completed before leaving the health facility.** If inconsistencies are detected (e.g., discrepancies in GA between visits), the following resolution process will be followed:

1. **Review of the data entry record** to determine if the issue is due to a data entry error.
2. If data entry is incorrect, the value will be corrected based on the source document.
3. If the discrepancy originates from the **source document**, the original value will be retained as-is and flagged.
4. **Unresolved inconsistencies** will be documented, flagged for further review, and evaluated in sensitivity analyses, if relevant.
5. Additional training or system adjustments will be implemented as needed to prevent recurrence of similar errors.
8.0 Data analysis

Data will be analyzed in accordance with the detailed procedures outlined in the **ANC Timing** **Data Analysis Plan**.

8.1 Statistical software

All data cleaning and analysis will be performed using **statistical software** such as **R studio**. Datasets will be cleaned and validated prior to analysis, and all analytic procedures will be documented in reproducible scripts, ensuring transparency and replicability of results.

8.2 Descriptive analysis

**Descriptive statistics** will be used to summarize all variables of interest. Measures such as counts, proportions, means, medians, standard deviations, and interquartile ranges will be calculated as appropriate for the distribution and type of variable.

The following types of **data visualizations** will be generated to support interpretation of findings:

- Frequency tables
- Histograms
- Bar charts
- Line graphs
- Box plots

Where applicable, data will be stratified by **level of care** (e.g., primary, secondary, tertiary). Other variables may be considered to allow patterns and disparities to be identified across subgroups and to support context-specific interpretation, such as:

- Participant age groups
- Facility type (e.g., public vs. private)
- Urbanicity
- ANC clinic volume

9.0 Acknowledgements

This protocol was developed by the PATH departments of Policy, Access and Introduction in the Center for Vaccine Innovation and Access and Maternal, Newborn Child Health & Nutrition with input from WHO’s Department of Immunization, Vaccines & Biologicals, Department of Maternal, Newborn Child and Adolescent Health and Ageing, and the Department of Sexual and Reproductive Health and Research. A special thank you to IS Global, the Pan American Health Organization, and Pasteur Institute in Nha Trang, Vietnam for pilot testing these tools and for reviews and input from the KEMRI-Wellcome Trust Research Programme in Kilifi, Kenya.

Supplementary Material 1b. Data analysis plan

**Characterizing the timing and attendance of antenatal care visits to inform the delivery of new maternal vaccines in low- and middle-income countries**

Generic ANC Timing Data Analysis Plan

PATH

17 November 2025

Table of Contents

[ANC Timing Data Analysis Plan introduction	3](#_Toc214871399)

[Clean data	3](#_Toc214871400)

[Define vaccine eligibility	4](#_Toc214871401)

[Define trimesters	4](#_Toc214871402)

[Data dictionary	5](#_Toc214871403)

[Table A. ANC Timing Data Dictionary.	5](#_Toc214871404)

[Estimate gestational age for each ANC visit	14](#_Toc214871405)

[Table B. Prioritization of variables to record and calculate gestational age at each ANC visit.	14](#_Toc214871406)

[Transform data	15](#_Toc214871407)

[Data analysis	16](#_Toc214871408)

[Exploratory data analysis	16](#_Toc214871409)

[Figure 1. Distribution of gestational age across antenatal care (ANC) visits.	16](#_Toc214871410)

[Primary study analyses	19](#_Toc214871411)

[Table 1. Characteristics of individuals attending ANC by level of care.	19](#_Toc214871412)

[Table 2. Individuals attending first ANC visit by trimester and level of care	20](#_Toc214871413)

[Figure 5. Mean gestational age at first ANC visit by number of ANC visits and level of care.	20](#_Toc214871414)

[Figure 6. Individuals attending antenatal care at least once by vaccine eligibility and level of care.	21](#_Toc214871415)

[Table 3. ANC visits per individual by level of care and trimester.	22](#_Toc214871416)

[Figure 7. Individuals attending ANC visits 1 through 8, aggregate.	23](#_Toc214871417)

[Figure 8. Gestational age in weeks by ANC visit, aggregate.	24](#_Toc214871418)

ANC Timing Data Analysis Plan introduction

This generic **ANC Timing** **Data Analysis Plan** is one part of a set of study materials that supports the conduct of a retrospective study entitled *Characterizing the timing and attendance of antenatal care visits to inform the delivery of new maternal vaccines in low- and middle-income countries*. The two other pieces of the set include a generic **ANC Timing** **Study Protocol** and a pre-programmed digital **ANC Timing** **Data Collection Tool,** which uses the open-source software, Kobo Toolbox.

This analysis plan provides guidance around cleaning and analyzing the study data once it has been collected. It includes a data dictionary, data transformations and cleaning steps for use with the **ANC Timing** **Data Collection Tool**, and the analyses required to address the study objective of characterizing the distribution of ANC visits by gestational age, along with example figures and tables. Designed generically for use in diverse settings, study investigators will need to adapt this analysis plan to align with local contexts.

The plan assumes that the source documents (i.e., ANC registers maintained at study health facilities) are paper-based and organized by ‘individual’ (i.e., all visits recorded for an individual’s index pregnancy are grouped together in the register) and that data follow the structure as described in the provided data dictionary. If registers differ, adjustments to the **ANC Timing Data Collection Tool** and corresponding updates to the analysis plan and data dictionary will be necessary. All analyses assume the use of the open-source statistical analysis program *R Studio*. Use of other analytical software may require modifications to data processing steps. To execute the analyses, the study team will need an analyst familiar with these software and descriptive statistical methods.

This **ANC Timing** **Data Analysis Plan** follows the following order of operations for data analysis:

- Clean data
- Define vaccine eligibility
- Define trimester
- Create and calculate variables using definitions in the data dictionary
- Prioritize gestational age variables
- Transform data so that each row of the data set represents the full ANC visit history of a specific individual
- Analyze data and generate tables and figures to show results

Clean data

After data collection is complete as per the **ANC Timing Study Protocol**, download the data from Kobo Toolbox to a file type compatible with the statistical software being used and proceed to data cleaning in the statistical software. Data cleaning includes removing unnecessary variables, formatting dates, and ensuring that data are in consistent units (e.g., ensuring pregnant individual ages are calculated to years and gestational ages are described in weeks).

**Review the dataset for potential errors** (e.g., implausible ages of the pregnant individual, missing data, or implausible gestational ages) and follow up with data collectors as needed to verify or correct entries. Articulate a plan to address the errors and apply it consistently. Keep a written record of decisions and relevant justifications.

**Screen for inconsistencies**. Articulate a plan to address the inconsistencies and apply it consistently. Keep a written record of decisions and relevant justifcations.

*EXAMPLE: Expectations are that gestational age should increase after each ANC visit for a pregnant individual. If gestational age decreases at subsequent visits, the investigator must decide how to use this data. One option is to use the data as written in the ANC register, even if it appears to be inaccurate. This would reflect the information that may be used by health providers at that visit to determine vaccine eligibility of an individual. Another option is to use the date and gestational age from previous visits, or other gestational age estimation methods (e.g., last menstrual period, estimated date of delivery) to calculate the gestational age more accurately.*

Define vaccine eligibility

Various vaccine eligibility gestational age ranges, or windows, can be explored in this study (e.g., 24–36 weeks; 32–36 weeks; ≥ 28 weeks); however, the primary definition should be selected in advance. World Health Organization Strategic Advisory Group of Expert on Immunization (WHO SAGE) position papers or national guidelines describing recommended administration timing of the relevant maternal vaccine are good sources to guide selection.

Define trimesters

Some recommendations for vaccine eligibility are set by trimester of pregnancy. Countries may use different definitions of trimester based on setting. Consult your country’s national guidelines for the thresholds of each trimester in defining your variables for vaccine eligibility and trimester categories. If there are no national definitions, consider using the Global Alignment of Immunization Safety Assessment in Pregnancy (GAIA) definitions of trimesters.^1^

^1^ GAIA NCIt Terminology Files: <https://evs.nci.nih.gov/ftp1/GAIA/About.html>

Data dictionary

The ANC Timing Data Dictionary for the study includes the variable definitions that will be used during data analysis (Table A). The pre-programmed variables from the ANC Timing Data Collection Tool are included in the data dictionary. Additional variables that must be created by the analysis team are described in the **Notes** column of Table A. If the investigator alters the variables collected in the Data Collection Tool, these changes must also be reflected in the data dictionary.

Table A. ANC Timing Data Dictionary.

| **Variable name in Kobo Toolbox** | **Common name** | **Definition** | **Variable type** | **Example** | **Notes** |
| --- | --- | --- | --- | --- | --- |
| Age related variables | | | | | |
| *age_recorded* | Age recorded | Indicates whether a pregnant individual’s age is documented in the ANC register. | Dichotomous (0,1) | 1 = Yes  0 = No |  |
| *age_format* | Format of Age variable | Indicates the format used to record the pregnant individual’s age in the register. | Categorical  (Birth year only, Birth date, Age, Category) |  |  |
| *age* | Age (years) | Age of the pregnant individual in years. | Integer | 30 | See “Transform Data” section to convert other Age Formats to *age.* |
| *birth_year* | Birth year | Birth year of the pregnant individual. | Integer | 1995 | See “Transform Data” section to convert other Age Formats to *age.* |
| *birth_date* | Birth date | Date of birth of the pregnant individual. | Calendar date (YYYY-MM-DD) | 1995-08-18 | See “Transform Data” section to convert other Age Formats to *age.* |
| *age_category* | Age category | Age group assigned to an indiviual when exact age is not recorded. | Categorical | 20–24 years | Use categories from your register. |
| ANC visit related variables | | | | | |
| *total_visits* | Total number of ANC visits | Total number of ANC visits an individual attended during the course of their pregnancy. | Integer | 5 = 5 total visits during pregnancy |  |
| *visit_number* | ANC visit number | Sequential order of an individual’s ANC visits during pregnancy. | Integer |  | When titling figures and tables, use: |
|  |  |  |  | 1 = First ANC visit in a pregnancy | ANC1 = First ANC visit in a pregnancy |
|  |  |  |  | 2 = Second ANC visit in a pregnancy | ANC2 = Second ANC visit in a pregnancy |
|  |  |  |  | 3 = Third ANC visit in a pregnancy | ANC3 = Third ANC visit in a pregnancy |
| *visit_date* | Date of ANC visit | Date an individual attended an ANC visit. | Calendar date (YYYY-MM-DD) |  | For analysis, data should be pivoted so each visit becomes a separate variable (visit_date1 = date of ANC1, visit_date2 = date of ANC2. |
| Gestational age related variables | | | | | |
| *edd_recorded* | Estimated date of delivery recorded | Indicates whether a pregnant individual’s estimated date of delivery is documented in the ANC register. | Dichotomous (0,1) | 1 = Yes  0 = No |  |
| *edd* | Estimated date of delivery | Date that birth is expected to occur. | Calendar date (YYYY-MM-DD) |  | See 'Estimate gestational age for each ANC visit’ section for guidance on converting estimated date of delivery to gestational age and prioritization of gestational age variables. |
| *fh_recorded* | Fundal height recorded | Indicates whether a pregnant individual’s fundal height is documented in the ANC register. | Dichotomous (0,1) | 1 = Yes  0 = No |  |
| *fh* | Fundal height | Fundal height measured at each ANC visit (cm=weeks). | Integer |  | For analysis, pivot data so each visit becomes a separate variable (e.g., fh1 = fundal height at ANC1, fh2 = fundal height at ANC2, etc.). |
|  |  |  |  |  | See ‘Estimate gestational age for each ANC visit’ section for guidance on converting fundal height to gestational age and prioritization of gestational age variables. |
| *ga_recorded* | Gestational age recorded | Indicates whether a pregnant individual’s gestational age is documented in the ANC register. | Dichotomous (0,1) | 1 = Yes  0 = No |  |
| *ga* | Gestational age | Number of completed weeks of pregnancy at the time of measurement rounded down to nearest whole week. | Integer | 0–6 days into week 20 is recorded as 20 weeks | For analysis, pivot data so each visit becomes a separate variable (e.g., ga1 = gestational age at ANC1, ga2 = gestational age at ANC2, etc.). |
|  |  |  |  |  | See 'Estimate gestational age for each ANC visit' section for guidance on prioritization of gestational age variables. |
| *lmp_recorded* | Last menstrual period recorded | Indicates whether a pregnant individual’s last menstrual period is documented in the ANC register. | Dichotomous (0,1) | 1 = Yes  0 = No |  |
| *lmp* | Last menstrual period | First day of the last menstrual period, used to estimate gestational age. | Calendar date (YYYY-MM-DD) |  | See 'Estimate gestational age for each ANC visit' section for guidance on converting last menstrual period to gestational age and prioritization of gestational age variables. |
| *us_recorded* | Ultrasound recorded | Indicates whether a pregnant individual’s ultrasound is documented in the ANC register. | Dichotomous (0,1) | 1 = Yes  0 = No |  |
| *us* | Ultrasound | Gestational age in weeks as estimated by ultrasound at the time of the scan, based on fetal measurements. | Integer |  | See 'Estimate gestational age for each ANC visit’ section for guidance on converting ultrasound to gestational age and prioritization of gestational age variables.  If variable is available, consider adding ultrasound date if ultrasound is used for estimating gestational age at subsequent visits. |
| *final_ga* | Final gestational age determination | The final gestational age for each visit to be used when multiple gestational age variables are available. | Integer |  | This variable will be created by the analysis team. See ‘Estimate gestational age for each ANC visit’ section for guidance on prioritization of gestational age variables. |
|  |  |  |  |  | For analysis, pivot data so each visit becomes a separate variable (e.g., final_ga1 = gestational age at ANC1, final_ga2 = gestational age at ANC2, etc.). |
| Facility characteristics | | | | | |
| *hf_name* | Health facility name | Name and identifier of the health facility. | Categorical |  | Names of eligible health facilities will be pre-populated into Kobo Toolbox. |
| *province* | Province | Province where the health facility is located. | Categorical |  | Names of provinces included in study will be pre-populated into Kobo Toolbox. |
| *district* | District | District where the health facility is located. | Categorical |  | Names of districts included in study will be pre-populated into Kobo Toolbox. |
| *town* | Town | Town where the health facility is located. | Categorical |  | Names of towns included in the study will be pre-populated into Kobo Toolbox. |
| *facility_type* | Type of facility | Indicates whether a health facility is public or private. | Categorical | Public  Private | Type of facility for each health facility included in the study will be pre-populated into Kobo Toolbox. |
| *loc* | Level of care | Indicates the level of care of a health facility. | Categorical | Primary  Secondary  Tertiary | Level of care for each health facility included in the study will be pre-populated into Kobo Toolbox. |
| *urbanicity* | Urbanicity | Indicates whether a health facility is located in an urban or rural area. | Categorical | Urban  Rural | Urbanicity for each health facility included in the study will be pre-populated into Kobo Toolbox. |
| *anc_clinic_volume* | ANC clinic volume | Indicates the volume of the ANC clinic based on number of women seen per month. | Categorical | Small  (1–100 women/month) | Use the administrative tallies at eligible health facilities to determine ANC clinic volume. Revise as needed as some facilities may record visits/month instead of women/month.  ANC clinic volume for each health facility included in the study will be pre-populated into Kobo Toolbox and inform the randomization sampling fraction. |
|  |  |  |  | Medium  (101–200 women/month) |  |
|  |  |  |  | Large  (> 200 women/ month) |  |
| Gestational age groups | | | | | |
| *trimester1* | Trimester 1 | Indicates whether an ANC visit occurred during the first trimester. | Dichotomous (0,1) | 1 = Visit occurred within first trimester  0 = Visit did not occur within first trimester | This variable will be created by the analysis team.  If using GAIA definitions of trimesters, use 1-13 weeks as the range. Otherwise, use your country’s guidelines for trimester definition. |
| *trimester2* | Trimester 2 | Indicates whether an ANC visit occurred during the second trimester. | Dichotomous (0,1) | 1 = Visit occurred within second trimester  0 = Visit did not occur within second trimester | This variable will be created by the analysis team.  If using GAIA definitions of trimesters, use 14–27 weeks as the range. Otherwise, use your country’s guidelines for trimester definition. |
| *trimester3* | Trimester 3 | Indicates whether an ANC visit occurred during the third trimester. | Dichotomous (0,1) | 1 = Visit occurred within third trimester  0 = Visit did not occur within third trimester | This variable will be created by the analysis team.  If using GAIA definitions of trimesters, use 28–42 weeks as the range. Otherwise, use your country’s guidelines for trimester definition. |
| *eligible* | Vaccine eligible | Indicates whether an ANC visit occurred during the gestational age window when an individual is eligible to receive a maternal vaccine. | Dichotomous (0,1) | 1 = Visit occurred within eligibility window  0 = Visit did not occur within eligibility window | This variable will be created by the analysis team.  If using WHO recommendation for eligibility, use the third trimester range in your local context (GAIA definition is 28–42 weeks). |
| *pre_eligible* | Before vaccine eligibility | Indicates whether an ANC visit occurred before the gestational age window for maternal vaccine eligibility. | Dichotomous (0,1) | 1 = Visit occurred before eligibility window  0 = Visit occurred during or after eligibility window | This variable will be created by the analysis team.  If using WHO recommendation for eligibility, use the first and second trimester range in your local context (GAIA definition is 1–27 weeks). |
| Other variables | | | | | |
| *reviewer_name* | Name of Data Collector | Name of the data collector who entered the information for this pregnant individual. | Categorical |  | This variable is used to help identify who to go to for questions if anything looks wrong in the data. |
| *uuid* | Unique ID | Unique identifier for the pregnant individual. | String |  | Auto-created by Kobo Toolbox for each entry (individual). |
| *index* | Row index | Unique identifier for each visit entered in the KoboToolbox form. | Number |  | This reflects the order in which visits were entered, not necessarily the chronological order of actual visits. |
| *parent_index* | Parent row index | A number that connects each visit to the individual it belongs to. | Number |  |  |

Estimate gestational age for each ANC visit

Key variables in this study are the gestational ages of pregnancy at each ANC visit. First, identify the variables recorded by your study facilities that may be used to indicate gestational age or to estimate it (Table B). Next, articulate a plan to prioritize the available variables to estimate gestational age at each visit based on completeness and accuracy of each variable. Note that this must be done *before* data analysis begins.

One example of variable prioritization is described in Table B. If the highest-priority option is not collected or is missing for that visit, move to the next option on the list. If any of the conditions are not met under ‘Conditions for using each variable’, move to the next option on the list.

Table B. Prioritization of variables to record and calculate gestational age at each ANC visit.

| **Priority** | **Variable (symbol in Kobo Toolbox tool)** | **Conditions for using each variable** | **Method to calculate gestational age** |
| --- | --- | --- | --- |
| 1 | Gestational age (*ga*) | Visit date must be available. | Record the gestational age (*ga)* in weeks as noted in the register for each visit. |
| 2 | Ultrasound (*us*) measure of gestational age | Gestational age at visit must be <13 weeks. | Record the gestational age in weeks associated with the ultrasound (*us)* as noted in the register for the given *visit_date.* |
| 3 | Last menstrual period (*lmp*) | Visit date must be available. | 1. Subtract date of visit (*visit_date)* from date of last menstrual period (*lmp*). 2. Divide by 7 (to get weeks). 3. Round down to nearest integer. |
| 4 | Estimated date of delivery (*edd*) | Visit date must be available. | 1. Subtract 280 days (average duration of a pregnancy) from estimated date of delivery (*edd)* (this approximates last menstrual period). 2. Subtract date of visit from date of last menstrual period approximation. 3. Divide by 7 (to get weeks) 4. Round down to nearest integer. |
| 5 | Fundal height (*fh*) | Gestational age at visit must be >20 weeks. | Fundal height in cm = weeks of gestation (i.e., 24 cm = 24 weeks). |

Using this priority order, the analyst should create a *final_ga* value for each visit of the pregnancy by selecting the highest-priority method available for measuring gestational age.

Transform data

**Objective**

Transform visit-level data so that each row in the dataset represents one individual.

**Step 1: Map data frames in statistical software to each sheet of data from the ANC Timing Data Collection Tool**

Start with two data frames (the two sheets of the data file downloaded from Kobo Toolbox).

Data frame 1 (df1) = Facility-level data and Individual-level data (one row per individual)

Data frame 2 (df2) = Visit-level data (one row per visit)

**Step 2: Remove unnecessary variables**

Remove columns that will not be needed for analysis, keeping only the variables in the definitions list.

Remove these columns:

- *end*
- *endtime*
- *id*
- *notes*
- *parent_table_name*
- *recordreviewdate*
- *start*
- *starttime*
- *status*
- *submission_id*
- *submission_notes*
- *submission_status*
- *submission_submission_time*
- *submission_submitted_by*
- *submission_tags*
- *submission_time*
- *submission_validation_status*
- *submission_version*
- *tags*
- *today*
- *validation_status*
- *version*

**Step 3: Organize and align visit-level data to be in chronological order of ANC visits for each individual**

Create a *visit_number* variable in both data frames.

Sort data by *uuid* and *visit_date*.

**Step 4: Transform the data to automate the re-organization of visit-level data to one row per individual**

Pivot the data in df2 to wide format. Each individual will have one row describing the gestational age at each visit with columns containing *ga1, ga2, ga3* for gestational ages at visits 1, 2, 3, etc.

**Step 5: Merge the data from both dataframes to have a complete dataset where one row represents the full ANC visit history and health facility information of a specific individual**

Merge the pivoted df2 with df1 by uuid to create one row that combines facility-level, individual-level and visit-level data for a given individual.

Data analysis

This section describes both exploratory analyses and primary analyses required to meet the study objective. The exploratory analyses allow the investigator to visualize the data and its distribution and inform the selection of appropriate descriptive statistics to use in the primary analyses (Figures 1–4). The primary analyses have seven key visualizations (tables or figures). The first three visualize the proportion of individuals attending ANC visits by trimester and characterize the gestational age at the first ANC visit (Table 1, Table 2, and Figure 5). Table 3 describes the number of ANC visits that individuals make across their pregnancies and identifies differences based on levels of care. Figure 6 (a key figure of the analysis) illustrates the proportion of individuals attending ANC before and during vaccine eligiblility. Figure 7 illustrates the proportion of individuals attending each ANC visit through the WHO recommended 8 ANC visits. Lastly, the other main figure of the primary analysis, Figure 8, demonstrates the distribution of gestational age at each ANC visit. This figure can help identify the ANC visit at which most individuals become eligible to receive a vaccine.

Exploratory data analysis

Start by viewing the data and selecting the descriptive statistics that work best for your context by creating a histogram of the gestational age across all ANC visits. If the distribution of gestational ages are normal, use the mean to describe the data. If the distribution of gestational ages are skewed, use the median. Consult a statistician to determine which descriptive statistics are most appropriate for your data.

Figure 1. Distribution of gestational age across antenatal care (ANC) visits.

Number of ANC Visits

| Gestational Age in Weeks | Series 1 |
| --- | --- |
| 0 | 0 |
| 1 | 10 |
| 2 | 10 |
| 3 | 10 |
| 4 | 10 |
| 5 | 10 |
| 6 | 20 |
| 7 | 20 |
| 8 | 20 |
| 9 | 20 |
| 10 | 20 |
| 11 | 25 |
| 12 | 30 |
| 13 | 40 |
| 14 | 50 |
| 15 | 60 |
| 16 | 70 |
| 17 | 80 |
| 18 | 90 |
| 19 | 100 |
| 20 | 120 |
| 21 | 120 |
| 22 | 112 |
| 23 | 114 |
| 24 | 100 |
| 25 | 95 |
| 26 | 90 |
| 27 | 85 |
| 28 | 80 |
| 29 | 70 |
| 30 | 60 |
| 31 | 50 |
| 32 | 40 |
| 33 | 30 |
| 34 | 20 |
| 35 | 20 |
| 36 | 20 |
| 37 | 10 |
| 38 | 10 |
| 39 | 10 |
| 40 | 10 |
| 41 | 5 |
| 42 | 2 |

Note: Each bar of the histogram reflects the number of ANC visits occurring at the given gestational age. This example bins gestational age with 1 week range to represent a single week distribution, but bins may be modified as needed. Individuals with multiple visits within that range will contribute multiple observations to that bar.

Additional exploratory analyses, such as those depicted in Figures 2–4, help the investigator visualize the data and may help in interpreting results from the primary analyses.

**Figure 2. Distribution of gestational age at first ANC visit.**

Number of Individuals

| Gestational Age in Weeks | Number of women |
| --- | --- |
| 0 | 0 |
| 1 | 3 |
| 2 | 5 |
| 3 | 5 |
| 4 | 5 |
| 5 | 7 |
| 6 | 8 |
| 7 | 10 |
| 8 | 25 |
| 9 | 27 |
| 10 | 30 |
| 11 | 35 |
| 12 | 37 |
| 13 | 40 |
| 14 | 45 |
| 15 | 35 |
| 16 | 40 |
| 17 | 50 |
| 18 | 55 |
| 19 | 53 |
| 20 | 55 |
| 21 | 60 |
| 22 | 62 |
| 23 | 65 |
| 24 | 68 |
| 25 | 75 |
| 26 | 73 |
| 27 | 80 |
| 28 | 75 |
| 29 | 70 |
| 30 | 65 |
| 31 | 70 |
| 32 | 90 |
| 33 | 88 |
| 34 | 85 |
| 35 | 88 |
| 36 | 90 |
| 37 | 70 |
| 38 | 50 |
| 39 | 30 |
| 40 | 10 |
| 41 | 5 |
| 42 | 3 |

**Figure 3. ANC visits completed per individual at study health facilities (%).**

Proportion of individuals

| Number of ANC Visits | Proprotion |
| --- | --- |
| 1 | 45% |
| 2 | 20% |
| 3 | 12% |
| 4 | 8% |
| 5 | 5% |
| 6 | 3% |
| 7 | 2% |
| 8 | 2% |
| 9 | 1% |
| 10 | 1% |
| 11 | 1% |
| 12 | 0% |

Note: This example includes a maximum of 12 ANC visits per person. The maximum should be modified to reflect the maximum number of ANC visits per person in the dataset used to create this chart. Note that the percentages should add up to 100%.

**Figure 4. Proportion of individuals attending at least one visit by trimester of pregnancy.**

Proportion of Individuals

|  | Proportion |
| --- | --- |
| Trimester 1 (1-13 weeks gestation) | 30% |
| Trimester 2 (14-27 weeks gestation) | 85% |
| Trimester 3 (28-42 weeks gestation) | 60% |

Note: In this example we used the GAIA definitions of trimester. Adjust the definitions as appropriate based on study context.

Primary study analyses

Table 1 illustrates the characteristics of the study population including how the number and proportion of individuals attending ANC within each trimester and describes the gestational age at the first ANC visit. Data are disaggregated by level of care to compare any differences in care-seeking behaviors.

Table 1. Characteristics of individuals attending ANC by level of care.

|  | **LEVEL OF CARE** | | | | | | | |
| --- | --- | --- | --- | --- | --- | --- | --- | --- |
|  | **PRIMARY** | | **SECONDARY** | | **TERTIARY** | | **ALL** | |
| **CHARACTERISTICS** | **N** | **%** | **N** | **%** | **N** | **%** | **N** | **%** |
| Facilities |  |  |  |  |  |  |  | 100.0 |
| Pregnant individuals |  |  |  |  |  |  |  | 100.0 |
| ANC visits |  | |  | |  | |  | |
| Mean age, years (SD) |  | |  | |  | |  | |
| **INDIVIDUALS WITH AT LEAST ONE VISIT WITHIN TRIMESTER** | | | | | | | | |
| Trimester 1 (1–13 weeks) |  |  |  |  |  |  |  |  |
| Trimester 2 (14–27 weeks) |  |  |  |  |  |  |  |  |
| Trimester 3 (28–42 weeks) |  |  |  |  |  |  |  |  |
| **GESTATIONAL AGE (WEEKS) AT FIRST ANC VISIT** | | | | | | | | |
| Mean (SD) |  | |  | |  | |  | |
| Median |  | |  | |  | |  | |
| IQR (Q1, Q3) |  | |  | |  | |  | |
| Minimum–Maximum |  | |  | |  | |  | |
| Definitions: ANC=antenatal care; IQR=interquartile range; N=number; %=percent; SD=standard deviation; Q1=first quartile; Q3=third quartile.  *Notes:*   - Global Alignment of Immunization Safety Assessment in Pregnancy (GAIA)^i^ definitions of trimester are used in this example. Substitute or supplement local definitions, as relevant. - Add additional time points of interest (e.g., 32–36 weeks gestation; 28–36 weeks gestation).   ^i^ GAIA NCIt Terminology Files: <https://evs.nci.nih.gov/ftp1/GAIA/About.html> | | | | | | | | |

Table 2 includes the proportion of pregnant individuals that attend their first ANC visit in the first, second, or third trimester by level of care. The data illustrate when individuals started their ANC visits and compare across levels of care.

Table 2. Individuals attending first ANC visit by trimester and level of care

|  | **LEVEL OF CARE** | | | | | | | | |
| --- | --- | --- | --- | --- | --- | --- | --- | --- | --- |
|  | **PRIMARY** | | **SECONDARY** | | **TERTIARY** | | | **ALL** | |
| **TRIMESTER** | **N** | **%** | **N** | **%** | **N** | **%** | **N** | | **%** |
| Trimester 1 (1–13 weeks) |  |  |  |  |  |  |  | |  |
| Trimester 2 (14–27 weeks) |  |  |  |  |  |  |  | |  |
| Trimester 3 (28–42 weeks) |  |  |  |  |  |  |  | |  |
| Definitions: ANC=antenatal care; N=number; %=percent of N. | | | | | | | | | |

*Note:* Global Alignment of Immunization Safety Assessment in Pregnancy (GAIA) definitions of trimester are used in this example. Substitute or supplement local definitions, as relevant.

Figure 5 describes the average gestational age of individuals at the first ANC visit by the total number of ANC visits made and any differences by level of care.

Figure 5. Mean gestational age at first ANC visit by number of ANC visits and level of care.

Gestational age in weeks

| Number of ANC visits per pregnancy | Primary Facilities | Secondary Facilities | Tertiary Facilities | All Facilities |
| --- | --- | --- | --- | --- |
| 1 | 34 | 25 | 38 | 29.6 |
| 2 | 28 | 24 | 36 | 25.8 |
| 3 | 27 | 23 | 20 | 22.7 |
| 4 | 20 | 22 | 15 | 19.3 |
| 5 | 18 | 20 | 15 | 18.4 |
| 6 | 16 | 16 | 14 | 15 |
| 7 | 14 | 12 | 15 | 13 |
| 8 | 13 | 10 | 15 | 12 |

*Note:* Mean gestational age is used for this figure, but median can be used if data is not normally distributed.

Figure 6 is a key figure in the analysis. It illustrates the proportion of individuals that attended ANC before eligibility for vaccination and when eligible for vaccination by level of care and aggregated across all levels of care. It highlights the proportion of individuals that have the opportunity to receive education and sensitization prior to vaccination at their ANC appointments, and the proportion of individuals that could potentially be vaccinated by providing the maternal vaccine during ANC visits.

Figure 6. Individuals attending antenatal care at least once by vaccine eligibility and level of care.

Proportion of individuals (%)

|  | Primary | Secondary | Tertiary | All |
| --- | --- | --- | --- | --- |
| Before vaccine eligibility (<28 weeks gestation) | 90% | 20% | 45% | 47% |
| Vaccine eligible (≥28 weeks gestation) | 85% | 85% | 70% | 75% |

*Notes:*

- In this example, vaccine eligibility is at the start of the third trimester, defined as 28 weeks gestation.
- Adjust the analysis according to local definintions of trimester or other vaccine eligibility criteria so visits are separated into *pre_eligible* and *eligible* groups.

*How to calculate the proportions:*

- Numerator: The number of individuals that had at least one ANC visit in the specific group (e.g., before vaccine eligibility or within vaccine eligibility window).
- Denominator: The total number of individuals at the given level of care.
- Calculation: Divide the numerator by the denominator to get the proportion (y axis).

Table 3 describes the average number of ANC visits per individual by level of care. This illustrates any differences in care seeking patterns by level of care. It also estimates the number of opportunities to either provide counseling about upcoming vaccine eligibility or to provide a vaccine when eligible.

|  | **ANC VISITS PER INDIVIDUAL** | | |
| --- | --- | --- | --- |
| **LEVEL OF CARE** | **MEAN (SD)** | **MEDIAN** | **MIN–MAX** |
| **PRIMARY** | | | |
| Before vaccine eligibility (<28 weeks) |  |  |  |
| Vaccine eligible (≥28 weeks) |  |  |  |
| **SECONDARY** | | | |
| Before vaccine eligibility (<28 weeks) |  |  |  |
| Vaccine eligible (≥28 weeks) |  |  |  |
| **TERTIARY** | | | |
| Before vaccine eligibility (<28 weeks) |  |  |  |
| Vaccine eligible (≥28 weeks) |  |  |  |
| **ALL** | | | |
| Before vaccine eligibility (<28 weeks) |  |  |  |
| Vaccine eligible (≥28 weeks) |  |  |  |

Table 3. ANC visits per individual by level of care and vaccine eligibility.

Definitions: ANC=antenatal care; Min**=**minimum; Max**=**maximum; SD=standard deviation.

Figure 7 depicts the proportion of individuals attending from one up to eight or more ANC visits. Note that the percentage of individuals with at least one ANC visit (the ANC1+ bar) will always be 100% since this is a criterium for study inclusion. This figure can be used to illustrate compliance with WHO recommendations for the number of ANC visits made in pregnancy.

Figure 7. Individuals attending ANC visits 1 through 8, aggregate.

Definitions: ANC=antenatal care; ANC1+=at least one ANC visit; ANC2+=at least two ANC visits, etc.

Proportion of individuals (%)

| ANC visit | All facilities |
| --- | --- |
| ANC1+ | 100% |
| ANC2+ | 95% |
| ANC3+ | 75% |
| ANC4+ | 60% |
| ANC5+ | 25% |
| ANC6+ | 15% |
| ANC7+ | 10% |
| ANC8+ | 5% |

*Note:* Create similar figures for all other categories, including levels of care and facility type (primary, secondary, tertiary, public facilities, private facilities, etc.).

*How to calculate each proportion:*

- Numerator: The number of individuals who had at least the number of visits for that category (e.g., all the individuals who had two or more visits for ANC2+)
- Denominator: The total number of individuals at the given level of care.
- Calculation: Divide the numerator by the denominator to get the proportion for that category. Repeat for all categories.

Figure 8 is the second key figure for the primary analysis. The box plot describes the gestational age of individuals by ANC visit. The dotted horizontal line marks the start of the vaccine eligibility window. The example in the figure uses vaccine eligibility at the third trimester and the GAIA third trimester definition starting at 28 weeks gestation. Each box plot shows the distribution of gestational age for that specific ANC visit, with the middle line of the box representing the median gestational age and the top and bottom of the box denoting the interquartile range; outliers are illustrated by dots. The percentage above each box is the proportion of individuals attending that ANC visit (per the example, 95% of individuals attended a second ANC visit [ANC2]). This figure can help identify the ANC visit at which most individuals were to receive a vaccine.

Figure 8. Gestational age in weeks by ANC visit, aggregate.

EXAMPLE

10%

15%

25%

60%

100%

95%

75%

21

36

38

10

28

30

34

35

5%

Definitions: ANC=antenatal care; ANC1=first ANC visit; ANC2=second ANC visits, etc.

*Note:* Similar figures can be created for specific categories of care (primary, secondary, tertiary, public facilities, private facilities, etc.).

1. WHO position paper on immunization to protect infants against respiratory syncytial virus disease, May 2025. https://www.who.int/publications/i/item/who-wer-10022-193-218
