# Supplementary material for: Timing and frequency of antenatal care visits in relation to maternal respiratory syncytial virus vaccine opportunities in four Latin American countries
Source: Vaccine. 2026 Aug 13;88:None. doi: 10.1016/j.vaccine.2026.128854 (PMC13481105; doi:10.1016/j.vaccine.2026.128854)
Supplement: Supplementary file 2 [file mmc2.docx]

**Supplementary Material 2**

Supplementary Table 1. Percentage of women attending antenatal care at least once before and during RSV vaccine eligibility windows, by regulatory authority and country.

|  | Before EMA [30] eligibility: < 24 weeks | Before WHO eligibility: < 28 weeks | Before PAHO eligibility: < 32 weeks | EMA eligible: 24–36 weeks | WHO eligible: ≥ 28 weeks | PAHO eligible: 32–36 weeks |
| --- | --- | --- | --- | --- | --- | --- |
| Bolivia | | | | | | |
| N | 3,544 | 3,902 | 4,132 | 4,215 | 4,216 | 3,514 |
| % | 81.2 | 89.4 | 94.7 | 96.6 | 96.6 | 80.5 |
| Ecuador | | | | | | |
| N | 3,635 | 4,027 | 4,368 | 3,635 | 4,372 | 3,692 |
| % | 76.8 | 85.1 | 92.3 | 76.8 | 92.4 | 78.1 |
| Honduras | | | | | | |
| N | 19,084 | 20,521 | 21,665 | 19,079 | 21,206 | 12,662 |
| % | 80.7 | 86.8 | 91.6 | 80.7 | 89.7 | 53.6 |
| Uruguay | | | | | | |
| N | 4,510 | 4,651 | 4,766 | 4,679 | 4,576 | 4,008 |
| % | 92.6 | 95.5 | 97.9 | 96.1 | 94.0 | 82.3 |
| Overall | | | | | | |
| N | 30,773 | 33,101 | 34,931 | 32,459 | 34,370 | 23,876 |
| % | 81.8 | 88.0 | 92.9 | 86.3 | 91.4 | 63.5 |

EMA = European Medicines Agency; PAHO = Pan American Health Organization; WHO = World Health Organization.

Supplementary Table 2. Percentage of women attending antenatal care at least once by trimester and country.

|  | Trimester 1:  1–13 weeks | Trimester 2:  14–27 weeks | Trimester 3:  28–44 weeks |
| --- | --- | --- | --- |
| Bolivia | | | |
| N | 2,166 | 3,830 | 4,216 |
| % | 49.6 | 87.8 | 96.6 |
| Ecuador | | | |
| N | 2,116 | 3,933 | 4,372 |
| % | 44.7 | 83.2 | 92.4 |
| Honduras | | | |
| N | 13,018 | 17,718 | 21,206 |
| % | 55.1 | 74.9 | 89.7 |
| Uruguay | | | |
| N | 3,646 | 4,553 | 4,576 |
| % | 74.9 | 93.5 | 94.0 |
| Overall | | | |
| N | 20,946 | 30,034 | 34,370 |
| % | 55.7 | 79.9 | 91.4 |
